# Retraction: Xu, et al. Treatment with Panax Ginseng Antagonizes the Estrogen Decline in Ovariectomized Mice. *Int. J. Mol. Sci.* 2014, *15*, 7827–7840

**DOI:** 10.3390/ijms21103461

**Published:** 2020-05-14

**Authors:** International Journal of Molecular Sciences Editorial Office

**Affiliations:** MDPI, St. Alban-Anlage 66, 4052 Basel, Switzerland; ijms@mdpi.com

It has been brought to our attention that this paper [1] was suspected of academic fraud. One stained tissue slice appears in both papers [1,2], but obviously it exposed two different situations of a mouse marked by immaturity and ovariectomy. The figure duplication brings uncertainty that the article is correct. Therefore, to ensure the addition of only high-quality scientific works to the field of scholarly publication, this paper [1] is retracted and shall be marked accordingly. MDPI is a member of the Committee on Publication Ethics and takes responsibility to enforce strict ethical policies and standards very seriously. We apologize to our readership that this went undetected until now.
